# Flexible metal-semiconductor-metal device prototype on wafer-scale thick boron nitride layers grown by MOVPE

**DOI:** 10.1038/s41598-017-00865-7

**Published:** 2017-04-11

**Authors:** Xin Li, Matthew B. Jordan, Taha Ayari, Suresh Sundaram, Youssef El Gmili, Saiful Alam, Muhbub Alam, Gilles Patriarche, Paul L. Voss, Jean Paul Salvestrini, Abdallah Ougazzaden

**Affiliations:** 1 UMI 2958, Georgia Tech - CNRS, 57070 Metz, France; 2 Georgia Institute of Technology, School of Electrical and Computer Engineering, GT-Lorraine, 57070 Metz, France; 3 CEA-LETI, Minatec Campus, F-38054 Grenoble, France; 4 Centre de Nanosciences et de Nanotechnologies, Université Paris-Saclay, C2N – Site de Marcoussis, route de Nozay, F-91460 Marcoussis, France; 5 Université de Lorraine, LMOPS, EA 4423, 57070 Metz, France

## Abstract

Practical boron nitride (BN) detector applications will require uniform materials over large surface area and thick BN layers. To report important progress toward these technological requirements, 1~2.5 µm-thick BN layers were grown on 2-inch sapphire substrates by metal-organic vapor phase epitaxy (MOVPE). The structural and optical properties were carefully characterized and discussed. The thick layers exhibited strong band-edge absorption near 215 nm. A highly oriented two-dimensional h-BN structure was formed at the film/sapphire interface, which permitted an effective exfoliation of the thick BN film onto other adhesive supports. And this structure resulted in a metal-semiconductor-metal (MSM) device prototype fabricated on BN membrane delaminating from the substrate. MSM photodiode prototype showed low dark current of 2 nA under 100 V, and 100 ± 20% photoconductivity yield for deep UV light illumination. These wafer-scale MOVPE-grown thick BN layers present great potential for the development of deep UV photodetection applications, and even for flexible (opto-) electronics in the future.

## Introduction

Hexagonal boron nitride (h-BN) has remarkable properties including a 2D graphene-analogue structure, high thermal conductivity, chemical inertness, strong mechanical strength, wide bandgap and large thermal neutron capture cross-section. It is a promising material for various electronic and optoelectronic devices. Two-dimensional h-BN layers are an ideal substrate and gate dielectric for graphene electronics or can be assembled with other 2D materials (van der Waals heterostructures) enabling the emergence of novel physical phenomena^1–6^. Few nanometer-thick h-BN layers can serve as the release layer for mechanical exfoliation of GaN-based device structures^7, 8^. Wafer-scale exfoliation was achieved^8^ based on the realization of large-area uniform 2D layered BN^9^.

BN can also be used for detector applications. With a large thermal neutron capture cross-section of around 3840 barns for B^10^, BN is a prominent candidate for neutron detectors used for the verification of nuclear materials and activities^10–13^. With a bandgap up to 6 eV, h-BN exhibits light absorption and emission in the UV-C spectral region^14–18^, leading to the potential for deep UV photodetectors for diverse applications including chemical, environmental and biological analysis for monitoring, flame and radiation detection, astronomical studies, and optical communications^19^. In the past, most of the BN-based photodetectors reported in the literature were based on boron nitride nanosheets (BNNS)^20–22^ or in BN monolayer domains^23^ with the size of micrometer scale for the usable surface area. The small size or non-uniformity of BN films would hinder its practical application in the industry fabrication. Besides, thicker BN films would be more favoured than monolayers or nanosheets to improve the detection yield. However, the structural properties of thick layers are unclear which require more investigation, and their application for photodetection is still at a very early stage^11–13^.

In this article, we report BN layers with thicknesses above 1 µm grown on 2-inch sapphire substrates by metal-organic vapor phase epitaxy (MOVPE). The structural and optical properties were explored, which is the basis for demonstrating applications of these grown crystal films. Metal-semiconductor-metal (MSM) device prototype fabricated on the BN membrane delaminated and separated from the substrate, which was possible because of the 2D layered structure at the film/substrate interface. The MSM deep UV photodiode prototypes demonstrated low dark current level of 2 nA under 100 V and strong photoconductivity yield of 100 ± 20% under deep UV light.

## Results and Discussions

**Figure 1:**
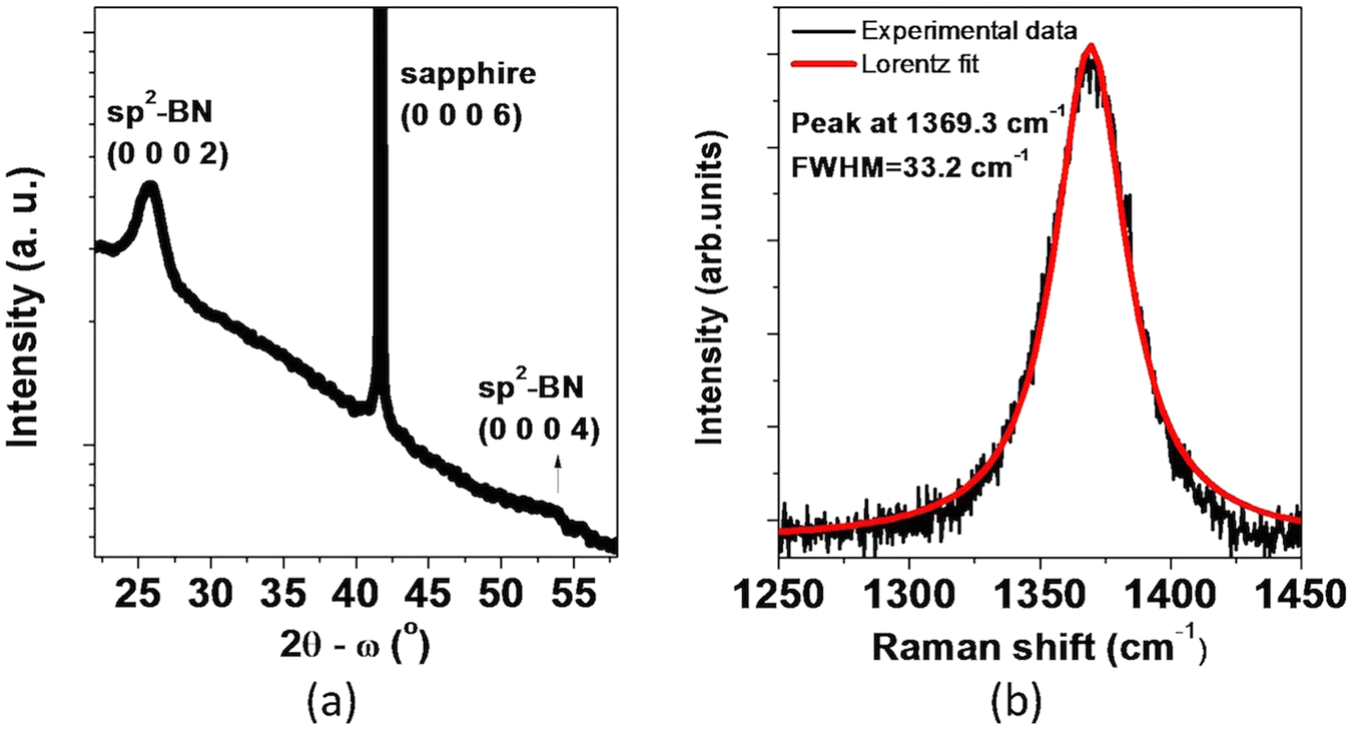
(**a**) High-resolution X-ray diffraction (HR-XRD) 2θ − ω scan; (**b**) Raman spectrum of BN layer grown on sapphire substrate.

Thick BN layers were grown by MOVPE on 2-inch sapphire substrates. The high resolution X-ray diffraction (HR-XRD) 2θ − ω scan of 1 µm BN on sapphire is presented in Fig. 1(a), in which a peak located at 25.8° is related to the (0 0 0 2) planes of the sp^2^-BN. A weak and broad peak corresponding to (0 0 0 4) planes was observed at around 53.1°. The Fig. 1(b) shows the Raman spectrum confirming the first-order E_2g_ mode of sp^2^-BN at 1369.3 cm^−1^ with a full-width at half maximum (FWHM) of 33.2 cm^−1^.

**Figure 2:**
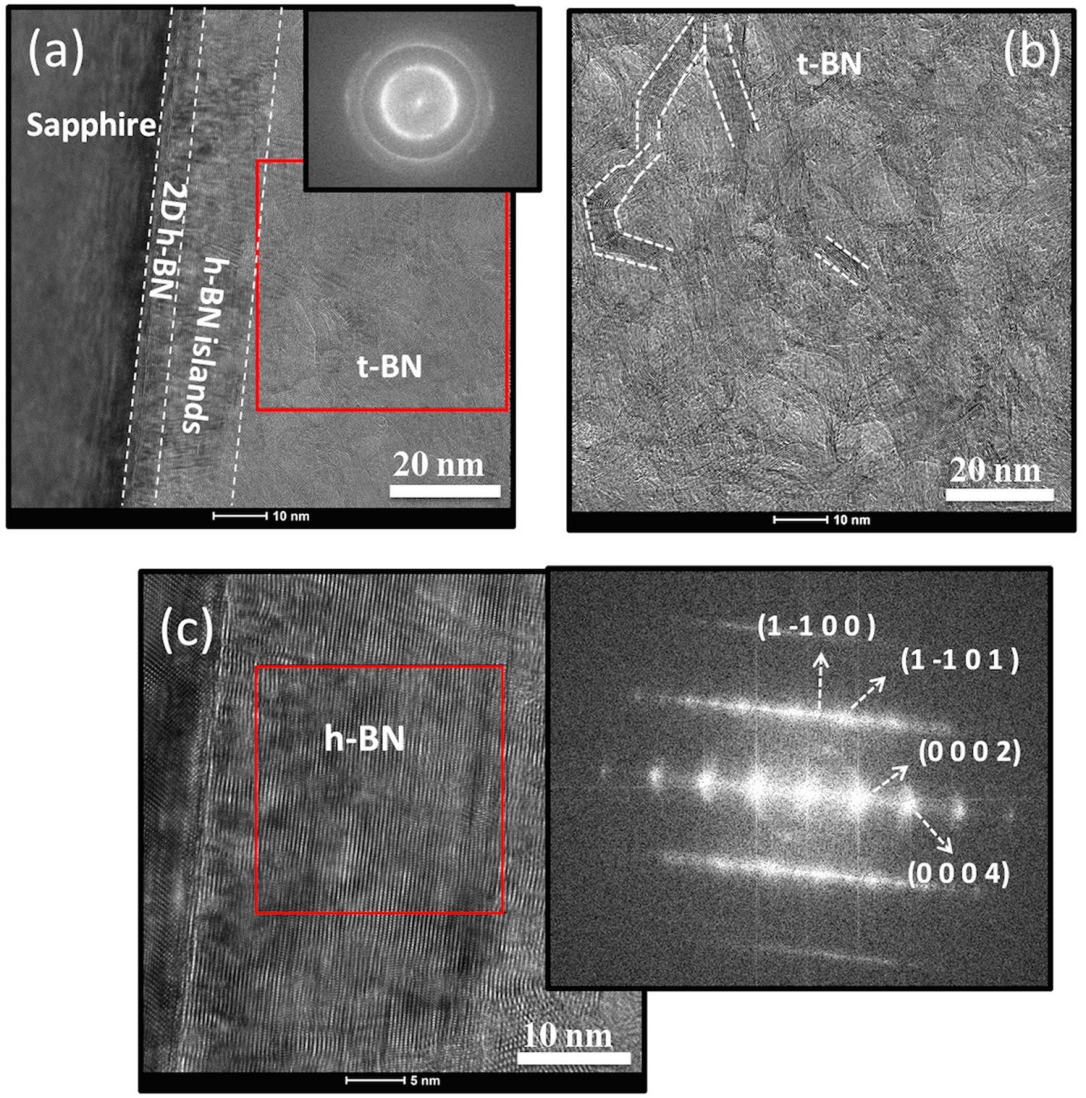
(**a**) Cross-section high-resolution TEM (HRTEM) image of 1 µm-thick BN grown on sapphire substrate which was taken along zone axis of <1 1–2 0>. Inset is the Fourier transform (FT) pattern for the selected area. (**b**) Higher magnification of the t-BN part showing randomly oriented BN nanofibers. (**c**) The initial layer near the substrate surface with the FT pattern in the inset confirming the hexagonal phase.

To elucidate the crystallographic properties of the grown thick sp^2^-BN, the cross-section high-resolution transmission electron microscopy (HRTEM) images were obtained and are shown in Fig. 2. In our previously published work^9^, 2D layered thin h-BN films with different thicknesses (up to 60 nm) were reported (by using low growth rate of 15 nm/h, details in Sample Preparation section). Structural and optical properties were studied. Especially, 3 nm h-BN showed a smooth surface, while hexagonal wrinkle pattern (a typical 2D morphology) was observed on the surface when the thickness was 30 nm, which was due to the compressive strain from cooling process. Wrinkle height was 5~10 nm for 30 nm h-BN, and it increased to 15~25 nm when the thickness of the layer was increased to 60 nm. Meanwhile, in this work, high growth rate of 470 nm/h was used to grow BN thick layers. With this condition, at the film/substrate interface, two-dimensional BN sheets with thickness of 5~6 nm formed. In the following 10~15 nm, stacking faults caused disordering of crystallographic planes, which hindered the growth of the uniform c-oriented layered lattice. The fast Fourier pattern of c-oriented region near the substrate (2D layered structure region) can be clearly identified as a hexagonal structure, as shown in the inset of Fig. 2(c). The following layer on the top part is turbostratic where the h-BN layered sheets are confined in short domains and irregularly oriented, losing long range order. This also explains the broadness of the sp^2^-BN peak in the XRD pattern in Fig. 1(a). The grains have a nanofiber feature^24^ as the examples labeled by the white lines in Fig. 2(b). These “fibers” have lengths ranging from few nanometers to several tens of nanometers.

**Figure 3:**
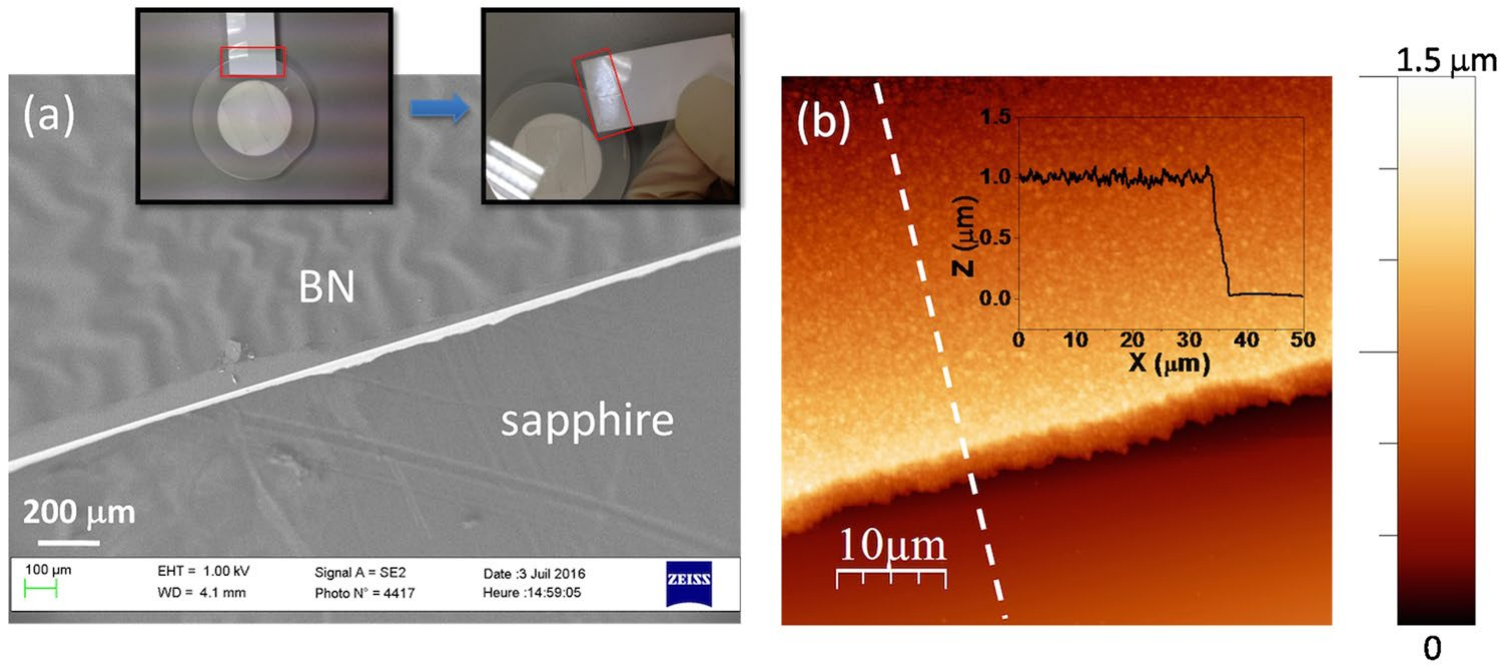
(**a**) Scanning electron microscopy (SEM) image focused at the edge where BN was lifted, and inset shows a lift-off and transfer process by simply using a scotch. (**b**) AFM image at the boundary with a height profile in the inset.

The uniform layered h-BN at the film/substrate interface enabled an excellent mechanical lift-off of the grown crystal layer by just using an adhesive support, since the weak van der Waals force between the atomic layers is easily broken. The inset of Fig. 3(a) demonstrates a simple process of lifting a targeted part of BN on the 2-inch wafer surface by using a commercial scotch. The scanning electron microscopy (SEM) image in Fig. 3(a) exhibits a sharp and straight ledge. The area where the BN was lifted presents a smooth morphology with root-mean square roughness as small as 0.27 nm. Ripples occurred in the neighboring BN part which was caused by the strain variation during exfoliation. The height profile in the AFM image in Fig. 3(b) shows that the step between the two areas is 1 µm, which indicates that the layer was mostly released from the bottom part of the BN film and transferred onto the adhesive sheet. This process can also be extended to the entire wafer as what has been done for several nanometer-thick BN in the previous report^8^. The effective process of release and transfer of thick BN films onto other support is fascinating for fabrication of low-cost flexible detection devices in the future.

**Figure 4:**
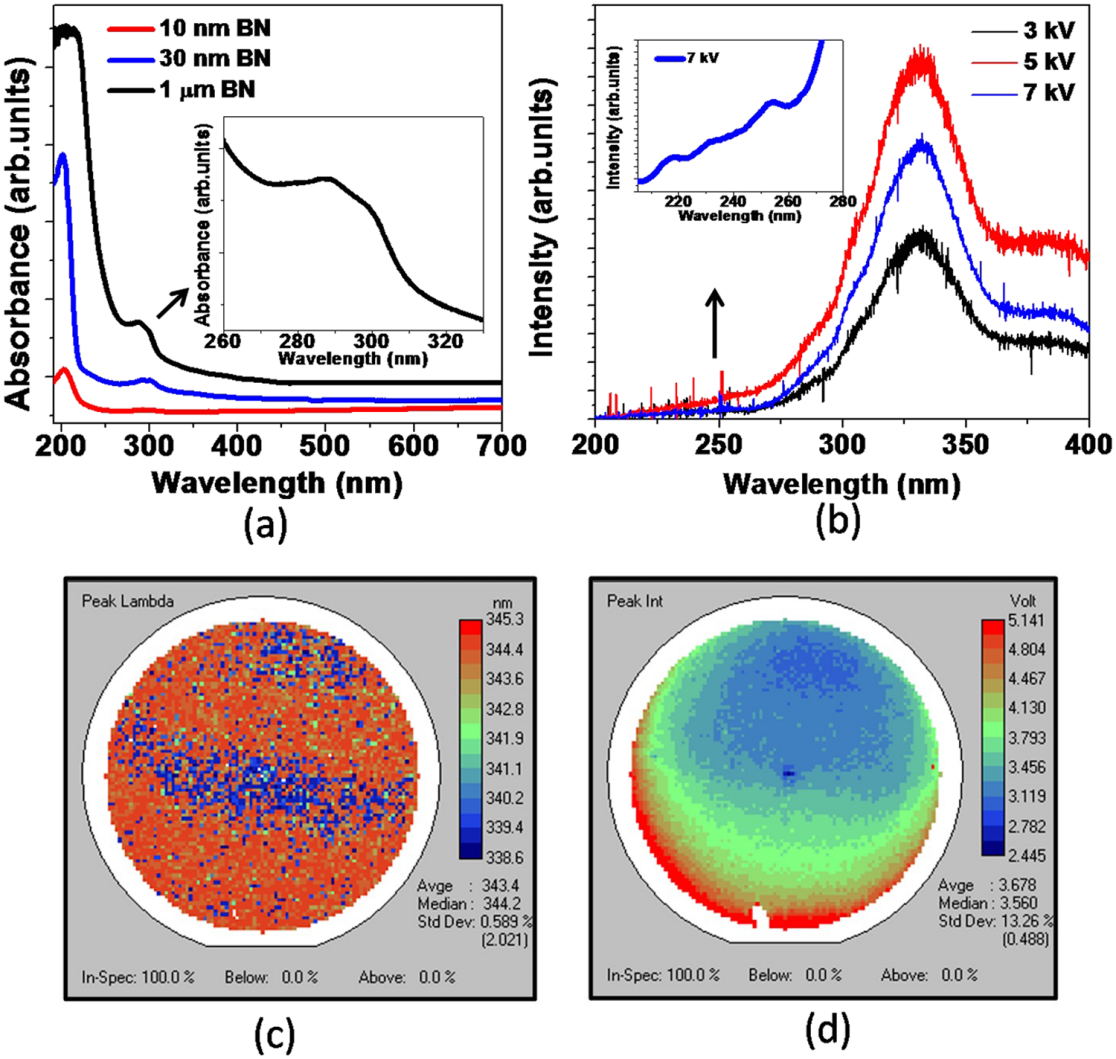
(**a**) Absorption spectrum of 1 µm BN grown on sapphire substrate compared with the absorption spectra of 10 nm and 30 nm 2D layered h-BN. Inset shows the absorption peaks related to defect levels. (**b**) Cathodoluminescence at 77 K under different excitation energy and inset shows the CL peaks in deep UV region. (**c**) Wavelength distribution and (**d**) intensity distribution of photoluminescence mapping performed at room temperature for 2-inch 1 µm BN/sapphire wafer.

To investigate optical properties of the grown thick BN layers, UV-Vis absorption tests were performed and the spectrum is shown in Fig. 4(a). For comparison, absorption spectra of 10 nm and 30 nm 2D layered h-BN were included, and they presented strong absorption peak at around 201 nm relating to π − π* interband transition^3, 25^. For 1 µm-thick BN layer, the absorption increases drastically for the wavelengths shorter than 250 nm and goes beyond detection limit below 215 nm (full absorption). Small absorption peaks at 288 nm and 299 nm are ascribed to the donor-acceptor pair transitions involving native defects in BN such as nitrogen vacancies (V_N_) and carbon impurities occupying the nitrogen sites (C_N_)^13, 26–28^.

Emission properties in UV region were examined as shown in Fig. 4(b). Cathodoluminescence (CL) at 77 K exhibits an intense and broad peak at around 332 nm, a characteristic deep-level band for this material relating to intrinsic point defects^13, 26–28^. As shown in the inset, there were multiple peaks in the short wavelength region similar to what was observed in 2D layered thin h-BN films^9^: at around 216 nm from near bandgap emission, at 232 nm and 253 nm from transitions involved in stacking faults^28^. Considering the huge absorption near the band edge at around 215 nm, the pronounced emission at 332 nm could be due to the exciton absorption or carriers transition to these midgap energy states. Figure 4(c) is the photoluminescence (PL) mapping performed at room temperature centered at this characteristic wavelength. Over the entire 2-inch wafer, the average emission wavelength is 343 nm which agrees with the CL results, and the standard deviation for the peak wavelengths is 0.6% and standard deviation of the peak intensity is 13% over the entire wafer surface.

**Figure 5:**
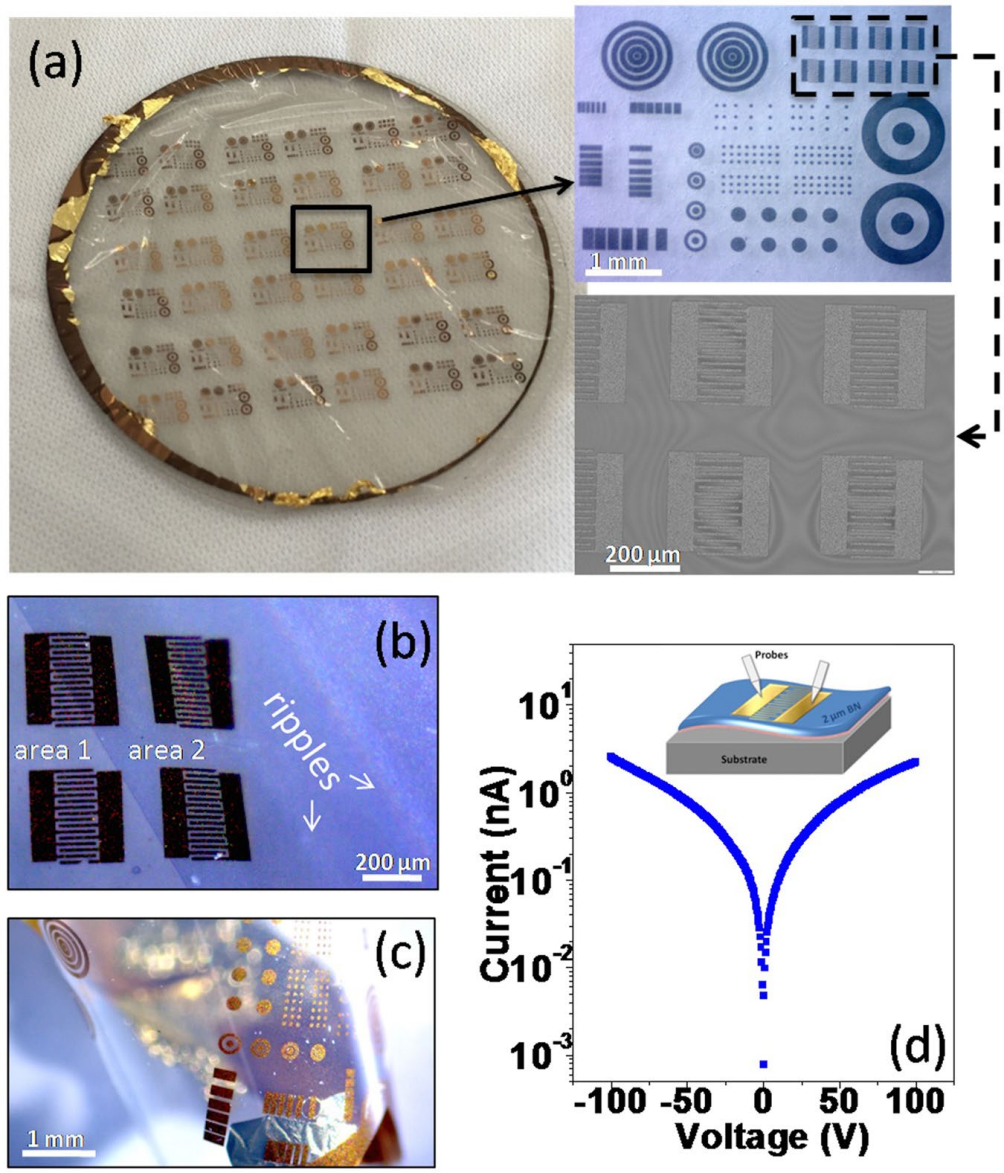
(**a**) BN wafer after device fabrication. Insets are microscope image of the metal contact patterns and scanning electron microscopy image of interdigitated electrodes. (**b**) Microscope image showing the local part where the film was separated from the substrate (area 2). (**c**) A small piece of freestanding BN prototype membrane lifted by tweezers which folded presenting the flexibility of the film. (**d**) Typical dark current-voltage (I-V) characteristics of the fabricated device prototypes and inset is the schematic of the measurements.

In order to investigate electrical characteristics and the photoresponse behavior of thick BN layers, the as-grown wafers of 2.5 µm BN were processed by deposition of electrodes that form MSM diodes with BN material. The interdigitated electrodes of Au have a width of 10 µm with spatial period of 20 µm. As one can see in Fig. 5(a), after processing, the grown film with devices features ripples exhibiting flexibility, with the center part adhered to the substrate. This is attributed to the special 2D layered structure of h-BN at the film/sapphire interface. It brings challenges for large-scale processing of this material, but at the same time, it provides opportunities to fabricate flexible semiconductor devices, as discussed previously.

The whole membrane maintained the structural integrity and is continuous without cracks or fractures despite the buckling and folding. Figure 5(b) is a local area where the film area 1 was still attached on the substrate and the area 2 was separated from the substrate with some ripples observed. It is noted that the straight boundary line between two areas came from the contrast difference and it is not a crack. A small piece of the freestanding BN membrane (square-millimetre scale) was lifted with tweezers shown in Fig. 5(c), and it instantaneously folded and rolled up several times without cracking demonstrating that this thick BN layer has good flexibility.

The schematic of the electrical measurements was shown in the inset of Fig. 5(d). The BN membrane with gold contact was curved with ripples and the probes were placed lightly on the contact. The typical current-voltage (I-V) curve measured on interdigitated patterns in the dark is shown in Fig. 5(d), which demonstrates symmetrical rectifying behaviour indicating back-to-back Schottky contacts. The diode exhibited very low dark current of around 2 nA under the applied voltage of 100 V.

**Figure 6:**
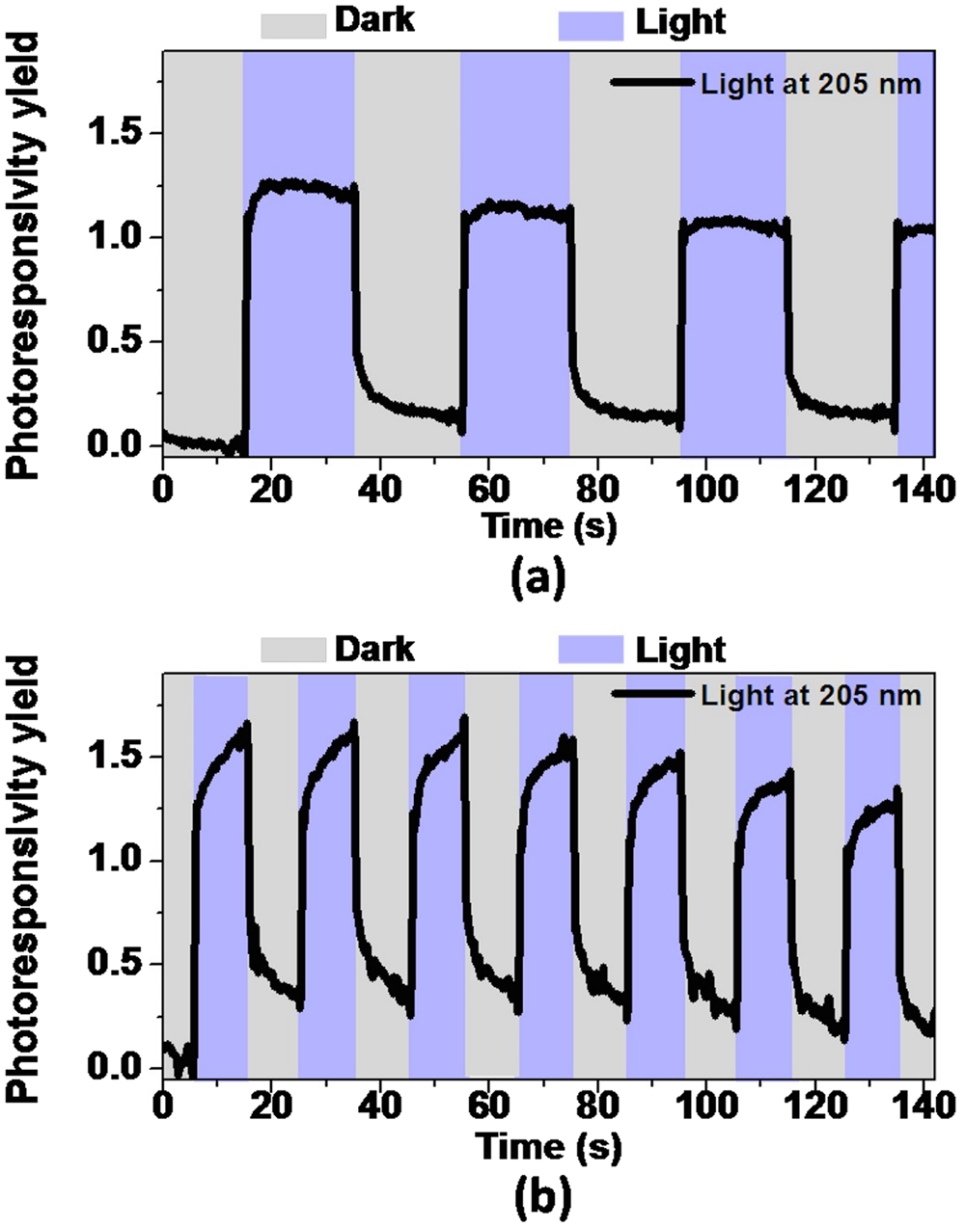
Response of prototypical thick BN-based photodetector (applied voltage of 100 V) under dark/light cycles (**a**) with pulse time of 20 s and (**b**) with pulse time of 10 s.

The fourth harmonic of a mode-locked Ti:Saphhire laser was focused on the device area to study the photoconductivity behavior of the fabricated sample at room temperature. The center wavelength of the ~150 fs pulses was 205 nm with estimated average intensity of 1 mW/mm^2^. Considering that a lateral MSM device structure on the surface was used for electrical measurements, that the deep UV light was illuminated from the top surface and that the BN layer has significant absorption at this wavelength, the t-BN region, which is much thicker than the bottom 2D layered region, is responsible for the performance and behavior of the prototype. Photoconductivity yield was calculated by Y_pc_ = (|I_light_ − I_dark_|)/I_dark_. As seen in Fig. 6 for dark/light pulses of 20 s and 10 s, at the moment when the light was switched on and off, the measured current has a steep change reaching a photoconductivity yield of around 100 ± 20%. Following that, a slow increase or decay component was observed which was caused by trapping/detrapping processes of the carriers at deep levels in the bandgap of the material^29–31^.

BN-based detectors reported in the literature showed very different performance^20–23^ and cannot be compared directly with each other due to the effect of material quality, contact configurations, applied voltages, UV light power densities and excitation wavelengths. Even the dark current level of BN or BNNS varied from hundred pA to several hundred nA due to different synthesis methods and processing techniques^13, 21–23^. But it is clear that the deep UV photodiode prototype fabricated here presented strong sensitivity and robust performance, with great potential for flexible devices as well.

## Conclusion

In summary, this work reports the wafer-scale growth of 1~2.5 µm-thick BN layers, deep structural and optical characterizations, and deep UV photodetector prototype demonstration. For the optical properties, the BN layers showed strong absorption below 215 nm and small absorption peaks at 288 nm and 299 nm from native defect levels. CL exhibited multiple peaks at around 216 nm, 232 nm and 253 nm from near band edge emission and stacking faults respectively, with a dominant characteristic defect band at 332 nm from mid-gap point defect states. Over the whole 2-inch wafer, there was only 0.6% wavelength variation and 13% intensity variation of the characteristic band emission by room temperature PL mapping. The crystallographic studies revealed that the BN layer had uniform two-dimensional layered structure at the film/substrate interface, which allows an effective mechanical exfoliation and transfer of the thick layer, and it also gives a photodiode prototype which delaminated from the substrate. This is of great interest for low-cost and flexible detection devices. The prototype exhibited low dark current level of around 2 nA under 100 V and photoconductivity yield of around 100 ± 20% under deep UV illumination at 205 nm. The progress achieved in this work opens the way to scaling up and low-cost production of detection applications based on wafer-scale growth of h-BN thick layers.

## Methods

### Sample preparation

Thick films of BN were grown on 2-inch sapphire substrates by MOVPE in an Aixtron close coupled showerhead (CCS) 3 × 2″ reactor. Triethylboron (TEB) and ammonia (NH_3_) were used as precursors for boron and nitrogen. The growth was performed at 1280 °C in hydrogen ambient at 85 mbar. TEB flow rate was 60 µmol/min. Previously, we have obtained 2D layered h-BN thin films (up to 60 nm) by using high NH_3_/TEB (V/III) ratio of 1000 and the growth rate was 15 nm/h^9^. In this work low V/III ratio of 52 was chosen in order to reduce growth time. The growth rate estimated from *in-situ* reflectance was as high as 470 nm/h. The thickness was also confirmed by cross-section scanning transmission electron microscopy images.

### Characterizations

High-resolution X-ray diffraction (HR-XRD) scans were done in Panalytical X’pert Pro MRD system with Cu Kα radiation. Raman shift spectra were measured by LabRam HR EVOLUTION Raman spectroscopy with laser excitation at 532 nm. For the crystallography studies, high-resolution transmission electron microscopy (HRTEM) characterizations were performed on Titan Themis microscope working at 200 kV and equipped with a Ceta 16 M camera. The cross-sectional sample was prepared using focused ion beam (FIB) thinning and ionmilling. 100 nm-thick carbon was deposited before FIB to protect the surface. Absorption measurements were done by a LAMDA 900 spectrometer. Emission properties were studied by cathodoluminescence (CL) at 77 K and photoluminescence (PL) mapping at room temperature.

### Wafer processing

The BN/sapphire wafers were cleaned using acetone and isopropanol followed by a dilute hydrochloric acid solution to remove organic residues and oxides prior to metallization. Standard photolithography was used to pattern the samples and 100 nm of Au was deposited by electron beam evaporation. The resulting structures were lifted off in a heated dimethyl sulfoxide (DMSO) solution, which were agitated for approximately 1 hour. During this step the thick BN membrane was partially separated from the substrate with the center part adhered to the substrate. Further gentle cleaning of the samples with acetone and isopropanol resulted in the final device structures used for electrical characterization.

### Laser characteristics

To test the photoresponse of the prototype, the fourth harmonic of a mode-locked Ti-Sapphire laser with ~150 fs pulses was used for the illumination. The wavelengths used were centered at 205 nm.
